# Oral Anticoagulants after Heart Transplantation—Comparison between Vitamin K Antagonists and Direct Oral Anticoagulants

**DOI:** 10.3390/jcm12134334

**Published:** 2023-06-28

**Authors:** Fabrice F. Darche, Lisa C. Fabricius, Matthias Helmschrott, Ann-Kathrin Rahm, Philipp Ehlermann, Tom Bruckner, Wiebke Sommer, Gregor Warnecke, Norbert Frey, Rasmus Rivinius

**Affiliations:** 1 Department of Cardiology, Angiology and Pneumology, Heidelberg University Hospital, 69120 Heidelberg, Germany; 2 Heidelberg Center for Heart Rhythm Disorders (HCR), Heidelberg University Hospital, 69120 Heidelberg, Germany; 3 German Center for Cardiovascular Research (DZHK), Partner Site Heidelberg/Mannheim, 69120 Heidelberg, Germany; 4 Institute for Medical Biometry, University of Heidelberg, 69120 Heidelberg, Germany; 5 Department of Cardiac Surgery, Heidelberg University Hospital, 69120 Heidelberg, Germany

**Keywords:** atrial fibrillation, bleeding, direct oral anticoagulant, heart transplantation, oral anticoagulant, stroke, vitamin K antagonist

## Abstract

Aims: Patients after heart transplantation (HTX) often require oral anticoagulants (OACs) due to atrial arrhythmias or thromboembolic events but little is known about the post-transplant use of direct oral anticoagulants (DOACs). We investigated the frequency, indications, and complications of DOACs and vitamin K antagonists (VKAs) after HTX. Methods: We screened all adult patients for the use of post-transplant OACs who underwent HTX at Heidelberg Heart Center between 2000 and 2021. Patients were stratified by type of OAC (DOAC or VKA) and by DOAC agents (apixaban, dabigatran, edoxaban, or rivaroxaban). Indications for OACs comprised atrial fibrillation, atrial flutter, pulmonary embolism, upper and lower extremity deep vein thrombosis, as well as intracardiac thrombus. Results: A total of 115 of 459 HTX recipients (25.1%) required OACs, including 60 patients with DOACs (52.2%) and 55 patients with VKAs (47.8%). Concerning DOACs, 28 patients were treated with rivaroxaban (46.7%), 27 patients with apixaban (45.0%), and 5 patients with edoxaban (8.3%). We found no significant differences between both groups concerning demographics, immunosuppressive drugs, concomitant medications, indications for OACs, ischemic stroke, thromboembolic events, or OAC-related death. Patients with DOACs after HTX had a significantly lower one-year rate of overall bleeding complications (*p* = 0.002) and a significantly lower one-year rate of gastrointestinal hemorrhage (*p* = 0.011) compared to patients with VKAs after HTX in the Kaplan–Meier estimator. Conclusions: DOACs were comparable to VKAs concerning the risk of ischemic stroke, thromboembolic events, or OAC-related death but were associated with significantly fewer bleeding complications in HTX recipients.

## 1. Introduction

Although heart transplantation (HTX) has been an established treatment for patients with end-stage heart failure for several decades, the clinical management of HTX recipients remains very challenging [1,2]. Various risk factors and complications can impair survival and quality of life after HTX including graft failure, acute rejection, infections, type 2 diabetes mellitus, heart rhythm disorders, and thromboembolic complications [3,4,5,6,7,8,9,10,11,12]. Particularly atrial fibrillation (AF), stroke, and venous thromboembolism (VTE) represent common causes of morbidity and mortality after HTX with reported overall incidences of 10.1% for AF, 10.7% for stroke, and 8.5% for VTE after HTX [10,11,12]. Given these numbers, oral anticoagulants (OACs) play an important role in the aftercare of HTX recipients but comprehensive guidelines for the use of OACs in HTX recipients are missing [1,13,14].

In terms of OACs, vitamin K antagonists (VKAs) were the primary OACs for several decades due to the absence of alternatives [13,14,15,16,17,18]. The disadvantages of VKAs are a long half-life, a narrow therapeutic window which requires constant laboratory monitoring, multiple drug-drug interactions, and prolonged re–establishment of the therapeutic window after a periprocedural pause [13,14,15,16,17,18,19]. During the last two decades, direct oral anticoagulants (DOACs) have been approved and clinically introduced which show a number of advantages over VKAs including a shorter half-life, no need for routine laboratory monitoring, fewer drug-drug interactions, and shorter periprocedural drug offset and onset effects [13,14,15,16,17,18,19]. In addition, several studies showed similar or even better efficacy and safety of DOACs over VKAs for the treatment of AF and VTE in the general population [20,21,22,23,24,25,26,27]. However, data on the efficacy and safety of DOACs in HTX recipients are very limited as they are often derived from small sample-size studies [17,18,19,28,29,30,31,32,33,34,35].

Given the little knowledge about the clinical management of HTX recipients requiring OACs, we decided to analyze HTX recipients with DOACs and VKAs focusing on indications and complications. In addition, we performed a sub-analysis of HTX recipients on DOACs comparing apixaban and rivaroxaban.

## 2. Patients and Methods

### 2.1. Patients

Our study was conducted in accordance with the ethical standards of the Declaration of Helsinki. The institutional review board (IRB) of Heidelberg University, Heidelberg, Germany, gave approval (ethics approval number: S-286/2015, Version 1.2, 28 July 2020). We obtained written informed consent from patients for their inclusion in the Heidelberg HTX Registry and the clinical and scientific use of their data. The ethics approval does not require additional consent for this observational study as only routine clinical data were utilized [4,5,6,7,8,9].

We screened all patients (≥18 years) for post-transplant use of OACs who underwent HTX at Heidelberg Heart Center, Heidelberg, Germany, between 2000 and 2021. Patients who had undergone repeat HTX were excluded. We also excluded patients with mechanical heart valves after HTX for comparison purposes as the use of DOACs is contraindicated in patients with mechanical heart valves [36]. All other adult patients with post-transplant use of OACs were included and stratified by OAC types (DOAC or VKA) and DOAC agents. Due to potential drug interactions with calcineurin inhibitors resulting in bleeding complications [13,14,34], the DOAC agent dabigatran was not used for HTX recipients at Heidelberg Heart Center. Besides this limitation, there was neither a preselection nor randomization of HTX recipients concerning the application of DOACs or VKAs during the study period as both agents were considered comparable, nor regarding the use of a specific DOAC agent (apixaban, edoxaban, or rivaroxaban). Factors influencing the prescription of DOACs or VKAs were individual physician’s practice and patient’s preference including pre-transplant use of DOACs or VKAs.

Indications for OACs in our study comprised AF, atrial flutter, pulmonary embolism, upper and lower extremity deep vein thrombosis (DVT), as well as intracardiac thrombus. There was no preselection or randomization of HTX recipients concerning the application of DOACs or VKAs during the study period as both agents were considered comparable.

### 2.2. Follow-Up

Follow-up of HTX recipients was performed in accordance with Heidelberg Heart Center’s routine clinical protocol. After hospital discharge following HTX, patients were seen monthly as outpatients in the HTX clinic during the first six post-transplant months, then bimonthly until the end of the first year after HTX, and approximately three to four times per year thereafter (with additional visits on demand) [4,5,6,7,8,9].

Post-transplant routine follow-up included medical history, physical examination, systolic and diastolic blood pressure measurement, blood and laboratory tests including immunosuppressive drug monitoring, resting 12-lead ECG, echocardiography, endomyocardial biopsy, annual chest X-ray as well as annual 24-h Holter monitor. We were able to obtain complete follow-up data after HTX from all patients as no patient was lost to follow-up [4,5,6,7,8,9].

### 2.3. Post-Transplant Medications

Medications after HTX including immunosuppressive drugs were administered as per Heidelberg Heart Center’s standard of care. Patients were perioperatively treated with an anti-thymocyte globulin-based immunosuppression induction therapy. The majority of patients in this study received an immunosuppressive drug therapy consisting of tacrolimus and mycophenolic acid as mycophenolic acid consequently replaced azathioprine from 2001 onward, and tacrolimus subsequently replaced cyclosporine A since 2006. In addition, everolimus was used depending on the clinical course of HTX recipients. Steroids were tapered incrementally during the initial post-transplant months and were routinely discontinued six months after HTX (unless clinically needed) [4,5,6,7,8,9].

### 2.4. Statistical Analysis

The primary outcome of this study was to compare overall bleeding complications between patients with DOACs or VKAs as oral anticoagulation after HTX. Causes of OAC-related bleeding complications after HTX were further assessed by stratification into the following categories: intracranial hemorrhage, severe epistaxis, gastrointestinal hemorrhage, and hemorrhagic shock. In addition, we analyzed the need for transfusion of FFP and PRBCs. Secondary outcomes included analysis of frequency and indications of OACs after HTX as well as ischemic stroke, thromboembolic events, and OAC-related death. We performed multiple univariate analyses in order to investigate potential intergroup differences between patients with DOACs or VKAs as oral anticoagulation after HTX as well as between patients with apixaban or rivaroxaban as oral anticoagulation after HTX. Analyzed variables comprised recipient data, recipient’s previous open-heart surgery, recipient principal diagnosis for HTX, donor data, transplant sex mismatch, perioperative data, immunosuppressive drug therapy, and post-transplant concomitant medications [4,5,6,7,8,9].

Data were analyzed using SAS (Version 9.4, SAS Institute, Cary, NC, USA) and shown as mean ± standard deviation (SD), median with quartiles (Q), or as count (*n*) with percentage (%). For measures of association, a difference of mean with a 95% confidence interval (CI) was applied. Depending on the variable type and question, we used Student’s *t*-test, Mann–Whitney U-test, analysis of variance (ANOVA), Kruskal–Wallis test, chi-squared test, or Fisher’s exact test, as appropriate. The Kaplan–Meier estimator using log-rank test was applied to graphically compare 1-year freedom from overall bleeding complications between patients with DOACs or VKAs as oral anticoagulation after HTX as well as to analyze 1-year freedom from gastrointestinal hemorrhage between patients with DOACs or VKAs as oral anticoagulation after HTX. A *p*-value of < 0.050 was considered statistically significant [4,5,6,7,8,9].

## 3. Results

### 3.1. Demographics of Heart Transplant Recipients with Oral Anticoagulants

After applying exclusion criteria, a total of 115 of 459 HTX recipients (25.1%) required the use of post-transplant oral anticoagulation, including 55 patients with VKAs (55 of 115 (47.8%)) and 60 patients with DOACs (60 of 115 (52.2%)). Concerning the 60 HTX recipients with DOACs, 27 patients were treated with apixaban (27 of 60 (45.0%)), 5 patients were treated with edoxaban (5 of 60 (8.3%)), and 28 patients were treated with rivaroxaban (28 of 60 (46.7%)). No patient received dabigatran (0 of 60 (0.0%)) due to potential interactions.

The median interval from HTX to the start of oral anticoagulation was 3.3 years (Q1: 0.3 years; Q3: 8.4 years) and the median interval from the start of oral anticoagulation until the end of oral anticoagulation was 0.8 years (Q1: 0.3 years; Q3: 2.3 years). There was neither a statistically significant difference between the median interval from HTX to the start of oral anticoagulation between patients with DOACs after HTX (3.5 years (Q1: 0.2 years; Q3: 9.3 years)) and patients with VKAs after HTX (3.3 years (Q1: 0.3 years; Q3: 8.1 years; *p* = 0.373)), nor a statistically significant difference between the median interval from the start of oral anticoagulation until the end of oral anticoagulation between patients with DOACs after HTX (0.8 years (Q1: 0.4 years; Q3: 2.4 years)) and patients with VKAs after HTX (0.7 years (Q1: 0.3 years; Q3: 2.3 years; *p* = 0.204)).

Concerning demographics, we found no statistically significant differences between patients with DOACs or VKAs after HTX with regard to recipient data, recipient previous open-heart surgery, recipient principal diagnosis for HTX, donor data, transplant sex mismatch, or perioperative data (all *p* ≥ 0.050). Demographics stratified by DOACs and VKAs after HTX are shown in Table 1.

Similarly, we observed no statistically significant differences between patients with apixaban or rivaroxaban after HTX relating to demographics (all *p* ≥ 0.050). Demographics stratified by apixaban and rivaroxaban after HTX are presented in Table 2.

### 3.2. Medications of Heart Transplant Recipients with Oral Anticoagulants

In terms of the immunosuppressive drug therapy, we discovered no statistically significant differences between patients with DOACs or VKAs after HTX regarding the use of cyclosporine A, tacrolimus, everolimus, azathioprine, mycophenolic acid, or steroids (all *p* ≥ 0.050).

We also observed no statistically significant differences between patients with DOACs or VKAs after HTX concerning the administration of oral antiplatelet drugs, beta-blockers, ivabradine, calcium channel blockers, angiotensin-converting-enzyme inhibitors/angiotensin II receptor blockers, diuretics, statins, or gastric protection drugs (all *p* ≥ 0.050). Medications stratified by DOACs and VKAs after HTX are provided in Table 3.

Likewise, there were no statistically significant differences between patients with apixaban or rivaroxaban after HTX concerning immunosuppressive drugs or concomitant medications (all *p* ≥ 0.050). Medications stratified by apixaban and rivaroxaban after HTX are given in Table 4.

### 3.3. Indications and Complications of Heart Transplant Recipients with Oral Anticoagulants

Indications for the use of OACs included 33 HTX recipients with post-transplant AF (28.7%), 27 HTX recipients with post-transplant atrial flutter (23.5%), 8 HTX recipients with post-transplant pulmonary embolism (7.0%), 12 HTX recipients with post-transplant upper extremity DVT (10.4%), 28 HTX recipients with post-transplant lower extremity DVT (24.3%), and 7 HTX recipients with post-transplant intracardiac thrombus (6.1%). 

We observed no statistically significant differences between HTX recipients with DOACs and VKAs regarding the indication of AF (*p* = 0.462), atrial flutter (*p* = 0.399), pulmonary embolism (*p* = 0.898), upper extremity DVT (*p* = 0.873), lower extremity DVT (*p* = 0.257), or intracardiac thrombus (*p* = 0.611).

Assessment of OAC-related complications showed no statistically significant differences between HTX recipients with DOACs and VKAs concerning ischemic stroke (*p* = 0.929), thromboembolic events (*p* = 0.611), or OAC-related death (*p* = 0.508) but HTX recipients with VKAs had a significantly higher percentage of overall bleedings (18 of 55 (32.7%)) in comparison to HTX recipients with DOACs (6 of 60 (10.0%); difference: 22.7%; 95% CI: 8.2–37.2%; *p* = 0.003). Indications and complications split by DOACs and VKAs after HTX are shown in Table 5.

In addition, Kaplan–Meier estimator displayed a significantly higher one-year rate of overall bleeding complications in patients with VKAs after HTX (*p* = 0.002). 

**Figure 1:**
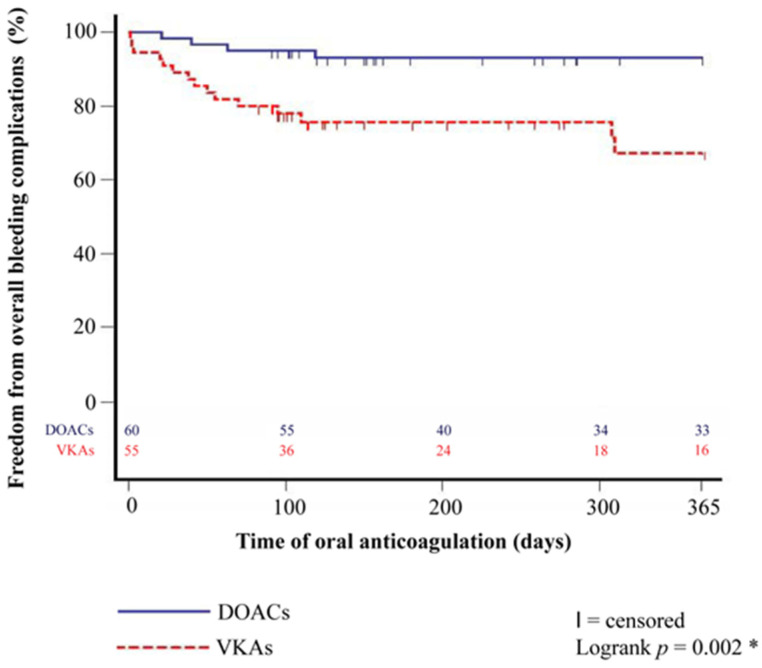
One-year freedom from overall bleeding complications between patients with DOACs and VKAs after HTX (Kaplan–Meier estimator). Patients with DOACs after HTX had a significantly lower one-year rate of overall bleeding complications than patients with VKAs after HTX (*p* = 0.002). DOAC = direct oral anticoagulant; HTX = heart transplantation; VKA = vitamin K antagonist; * = statistically significant (*p* < 0.050).

**Figure 2:**
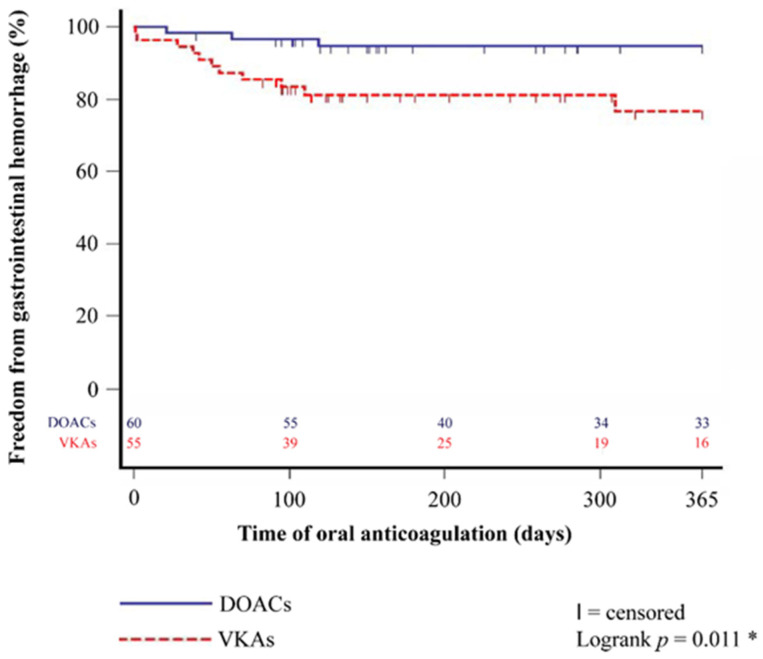
One-year freedom from gastrointestinal hemorrhage between patients with DOACs and VKAs after HTX (Kaplan–Meier estimator). Patients with DOACs after HTX had a significantly lower one-year rate of gastrointestinal hemorrhage than patients with VKAs after HTX (*p* = 0.011). DOAC = direct oral anticoagulant; HTX = heart transplantation; VKA = vitamin K antagonist; * = statistically significant (*p* < 0.050).

Further investigations revealed that HTX recipients with VKAs showed a significantly higher percentage of gastrointestinal hemorrhage (12 of 55 (21.8%) vs. 4 of 60 (6.7%); difference: 15.1%; 95% CI: 2.5–27.7%; *p* = 0.019) and required more frequent transfusion of PRBCs (16 of 55 (29.1%) vs. 6 of 60 (10.0%); difference: 19.1%; 95% CI: 4.9–33.3%; *p* = 0.009). Patients with VKAs after HTX also had a higher one-year rate of gastrointestinal hemorrhage in the Kaplan–Meier estimator (*p* = 0.011). Kaplan–Meier estimators are displayed in Figure 1 and Figure 2.

At the time of bleeding complications, two-thirds of HTX recipients with VKAs (12 of 18 (66.7%)) had an international normalized ratio (INR) level above the therapeutic range which is associated with a higher risk of bleeding. In contrast, we could not observe a relationship between DOAC dosing and bleeding complications of those six HTX recipients on DOACs who suffered from bleeding complications, only two patients were on full dose DOACs (2 of 6 (33.3%)), while four patients were on reduced dose DOACs (4 of 6 (66.7%)).

In terms of dosing of DOACs in general, 30 of 60 HTX recipients (50.0%) received a reduced dose of DOAC. Reasons for dose adjustment included reduced renal function in 20 of 30 HTX recipients (66.7%) and concomitant anti-platelet use in 10 of 30 HTX recipients (33.3%). Comparison of HTX recipients with apixaban or rivaroxaban showed no statistically significant difference in overall reduced dose of DOAC (13 of 27 (48.1%) vs. 12 of 28 (42.9%); difference: 5.2%; 95% CI: −21.1–31.5%; *p* = 0.694), dose adjustment of DOAC due to reduced renal function (8 of 27 (29.6%) vs. 7 of 28 (25.0%); difference: 4.6%; 95% CI: −18.9–28.1%; *p* = 0.700), or a dose of DOAC adjustment due to concomitant anti-platelet use (5 of 27 (18.5%) vs. 5 of 28 (17.9%); difference: 0.6%; 95% CI: −19.8–21.0%; *p* = 0.949). We also observed no statistically significant differences between HTX recipients with apixaban or rivaroxaban in terms of indications, OAC-related complications, and OAC-related bleeding (all *p* ≥ 0.050). Indications and complications split by apixaban and rivaroxaban after HTX are given in Table 6.

### 3.4. Sensitivity Analysis

Due to the long study period (2000–2021), we investigated a possible era effect by dividing all 115 HTX recipients with OACs into two different time periods (48 patients with the date of HTX between 2000 and 2009 vs. 67 patients with the date of HTX between 2010 and 2021). There was no statistically significant difference regarding the use of OACs between patients who received HTX between 2000 and 2009 (27 of 48 HTX recipients with VKA (56.2%) vs. 21 of 48 HTX recipients with DOACs (43.8%)) and patients who received HTX between 2010 and 2021 (28 of 67 HTX recipients with VKA (41.8%) vs. 39 of 67 HTX recipients with DOACs (58.2%); *p* = 0.126). Further analysis showed comparable results for both subgroups supporting the robustness of our findings and reducing the likelihood of a potential era effect.

## 4. Discussion

### 4.1. Frequency and Indications of Oral Anticoagulants after Heart Transplantation

Clinical management of HTX recipients frequently involves the treatment of atrial arrhythmias or thromboembolic events implying the need for OACs [10,11,12,13,14,15,16]. However, data about OACs in HTX recipients, especially about the efficacy and safety of DOACs, are scarce and mainly based on case series or small sample size studies [17,18,19,28,29,30,31,32,33,34,35]. We, therefore, performed the largest known study about the frequency, indications, and complications of DOACs after HTX. A total of 115 of 459 HTX recipients (25.1%) required OACs, including 60 patients with DOACs (52.2%) and 55 patients with VKAs (47.8%). This frequency of OACs after HTX is in line with findings by Tremblay-Gravel and colleagues [35] who reported 80 of 426 HTX recipients (18.8%) on OACs, including 57 patients with DOACs (71.3%), as well as with findings by Kim and colleagues [33] who reported 18 of 55 HTX recipients (32.7%) on OACs, including 7 patients with DOACs (38.9%).

Among HTX recipients with DOACs, most patients in our study received either apixaban (45.0%) or rivaroxaban (46.7%), while only a minority received edoxaban (8.3%). Given the potential interactions with calcineurin inhibitors resulting in bleeding complications [13,14,34], no patient in our study received dabigatran. A similar distribution of DOACs after HTX was reported by Bellam and colleagues [32] with apixaban (73.9%) and rivaroxaban (26.1%) as the two most used DOACs, while also no patient received dabigatran.

As DOACs can be used for several indications [13,14], we compared the different indications of DOACs and VKAs after HTX. We found no significant differences between HTX recipients with DOACs or VKAs concerning the indications of AF, atrial flutter, pulmonary embolism, upper extremity DVT, lower extremity DVT, and intracardiac thrombus. We would like to emphasize that we excluded patients with mechanical heart valves after HTX for comparison purposes as the use of DOACs is contraindicated in patients with mechanical heart valves [36]. 

Altogether, about one-quarter of HTX recipients in our study required OACs for several indications, highlighting the clinical importance of DOACs as an alternative to VKAs.

### 4.2. Efficacy of Oral Anticoagulants after Heart Transplantation

The primary goal of OACs is the prevention of thromboembolic stroke in patients with AF and the prevention of the progression or recurrence of thromboembolic events in patients with VTE [13,14,15,16]. Several studies have shown a comparable efficacy of DOACs in comparison to VKAs in the general population [20,21,22,23,24,25,26,27] but data about the efficacy of DOACs after HTX are limited [17,18,19,28,29,30,31,32,33,34,35].

In terms of efficacy, we observed no statistically significant differences between HTX recipients with DOACs and VKAs concerning ischemic stroke (3.3% vs. 3.6%), thromboembolic events (3.3% vs. 1.8%), or OAC-related death (1.7% vs. 3.6%). Similar results were reported by Henricksen and colleagues [19] who reported VTE recurrence in 2 of 51 HTX recipients with DOACs (3.9%), while they observed no recurrence of VTE in 22 HTX recipients with VKAs (0.0%). Likewise, Lichvar and colleagues [28] reported two VTE (5.4%) during DOAC therapy in 37 cardiothoracic transplant recipients including five patients with HTX, one single lung transplant recipient with lower extremity VTE, and one HTX recipient with a left ventricular apical thrombus. In addition, no strokes or transient ischemic attacks were reported [28].

Regarding the efficacy of apixaban and rivaroxaban in HTX recipients, we detected no statistically significant differences concerning ischemic stroke, thromboembolic events, or OAC-related death which is in accordance with results by Pasley and colleagues [29] who also reported no statistically significant differences between 26 cardiothoracic transplant recipients with apixaban and 12 cardiothoracic transplant recipients with non-apixaban DOACs (10 patients with rivaroxaban and 2 patients with dabigatran) regarding thromboembolic events (*p* = 0.23) or death while on DOAC (*p* = 1.0).

In this light, the above-mentioned data suggest that DOACs are as effective as VKAs in HTX recipients regarding the prevention of ischemic stroke and VTE after HTX. In addition, the efficacy of apixaban and rivaroxaban in HTX recipients appears to be comparable.

### 4.3. Safety of Oral Anticoagulants after Heart Transplantation

Besides efficacy, safety plays an important role in HTX recipients requiring OACs [13,14]. The safe use of VKAs necessitates a stable therapeutic INR level within a narrow therapeutic window including close laboratory monitoring [13,14,37,38,39]. Lower INR levels can increase the risk of thromboembolic events, while INR levels above the therapeutic range are associated with a higher risk of bleeding complications [13,14,37,38,39,40,41,42,43]. A time in the therapeutic range (TTR) > 70% is regarded as INR stability [40]. However, this target TTR is rarely achieved or sustained for long [41]. In terms of patients after HTX, there are no available data about the percentage of time in which HTX recipients with VKAs are in the therapeutic range but Pokorney and colleagues [42] reported that patients in community-based clinical practice with AF and VKAs had INR levels between 2.0 and 3.0 only in 59% of the time. Likewise, Rose and colleagues [43] reported a rate of only 58% of INR levels in the therapeutic range. The causes for INR instability are multifactorial including age, heart failure, diabetes mellitus, alimentation, adherence to therapy, drug interactions, and genetic polymorphisms which makes it difficult to predict future changes in INR levels [37,38,39,40,41,42,43]. Thus, clinical prediction tools can only explain less than 10% of INR fluctuations and more than 40% of all hemorrhagic events occur at INR levels > 3.0 [41].

In our study, HTX recipients with VKAs had a significantly higher percentage of overall bleeding complications, gastrointestinal hemorrhage, and transfusion of PRBCs in comparison to HTX recipients with DOACs. Of notice, two-thirds of HTX recipients with VKAs who suffered from overall bleeding complications had an INR level above the therapeutic range (12 of 18 (66.7%)). Similar results were reported by Henricksen and colleagues [19] who observed a trend toward a lower rate of overall bleeding complications in HTX recipients with DOACs (5 of 51 (9.8%)) compared to HTX recipients with VKAs (5 of 22 (22.7%); *p* = 0.08). Furthermore, they found a significantly lower rate of bleeding requiring transfusion in HTX recipients with DOACs (*p* = 0.04) compared to HTX recipients with VKAs [19].

Concerning the safety of apixaban and rivaroxaban in HTX recipients, we observed no statistically significant differences regarding overall bleeding complications, gastrointestinal hemorrhage, or transfusion of PRBCs which is in line with findings by Pasley and colleagues [29] who also found no statistically significant differences between 26 cardiothoracic transplant recipients with apixaban and 12 cardiothoracic transplant recipients with non-apixaban DOACs (ten patients with rivaroxaban and two patients with dabigatran) regarding overall bleeding complications (*p* = 0.35).

Hence, based on our data and the findings from other studies, the use of DOACs after HTX appears safe and effective. Given the lack of data about the use of edoxaban after HTX and the potential interactions between dabigatran and calcineurin inhibitors, apixaban or rivaroxaban seem to be the first choice for the treatment of atrial arrhythmias or thromboembolic events after HTX.

### 4.4. Study Limitations

Our findings were derived from a large single-center registry (Heidelberg HTX Registry). Given the known limitations of this retrospective analysis of data, our findings should be interpreted carefully and within the context of the existing literature. However, we would like to emphasize that our analysis is the largest known study so far about the use of OACs in HTX recipients comparing DOACs and VKAs. Furthermore, we obtained highly detailed data from all 115 HTX recipients with OACs, as our patients received standardized treatment and follow-up, reducing the likelihood of selection bias and potential confounders [4,5,6,7,8,9].

In order to acquire a reasonable number of HTX recipients with post-transplant use of OACs, we decided to analyze patients who received HTX at the Heidelberg Heart Center between 2000 and 2021. Given the long study period, a possible era effect due to changes in surgical and medical care may have influenced our results. We, therefore, investigated a possible era effect by dividing HTX recipients with OACs into two different time periods. We found no statistically significant difference regarding the use of OACs between patients who received HTX between 2000 and 2009 vs. 2010 and 2021. In addition, a sensitivity analysis of both groups showed similar findings supporting the robustness of our results and reducing the likelihood of a potential era effect [4,5,6,7,8,9].

Given the lack of routine assessment of DOAC-specific anti-Xa activity, we could not perform further investigations to explore the use of DOAC-specific anti-Xa monitoring. However, data about the benefits of DOAC-specific anti-Xa monitoring in HTX recipients are rare and its clinical use is still the subject of debate [19,30,31].

Finally, our findings should be interpreted as hypothesis-generating, particularly in the context of bleeding complications after HTX as several factors can cause an increased risk for hemorrhage. We can therefore neither prove nor disprove a causal relationship but merely indicate an association. Additionally, long-term differences between DOACs and VKAs in HTX recipients remain unknown and require further investigation, preferably in the form of large multicenter trials.

## 5. Conclusions

In summary, based on our results, DOACs were comparable to VKAs concerning the risk of ischemic stroke, thromboembolic events, or OAC-related death but were associated with significantly fewer bleeding complications in HTX recipients. In addition, subgroup analysis of HTX recipients with apixaban and rivaroxaban indicated comparable effects of both agents regarding clinical efficacy and safety after HTX.

## Figures and Tables

**Table 1:** Demographics—stratified by DOACs and VKAs after HTX.

Parameter	All OACs after HTX (*n* = 115)	DOACs after HTX (*n* = 60)	VKAs after HTX (*n* = 55)	Difference	95% CI	*p*-Value
Recipient data						
Age (years), mean ± SD	52.4 ± 10.4	52.0 ± 10.8	52.9 ± 9.9	0.9	−2.9–4.7	0.652
Male sex, *n* (%)	84 (73.0%)	44 (73.3%)	40 (72.7%)	0.6%	−15.6–16.8%	0.942
BMI (kg/m^2^), mean ± SD	25.2 ± 4.1	25.1 ± 4.2	25.4 ± 4.0	0.3	−1.2–1.8	0.678
Arterial hypertension, *n* (%)	61 (53.0%)	28 (46.7%)	33 (60.0%)	13.3%	−4.8–31.4%	0.152
Dyslipidemia, *n* (%)	69 (60.0%)	32 (53.3%)	37 (67.3%)	14.0%	−3.7–31.7%	0.127
Diabetes mellitus, *n* (%)	26 (22.6%)	11 (18.3%)	15 (27.3%)	9.0%	−6.3–24.3%	0.252
Peripheral artery disease, *n* (%)	4 (3.5%)	2 (3.3%)	2 (3.6%)	0.3%	−6.4–7.0%	0.929
COPD, *n* (%)	14 (12.2%)	7 (11.7%)	7 (12.7%)	1.0%	−11.0–13.0%	0.862
History of smoking, *n* (%)	56 (48.7%)	32 (53.3%)	24 (43.6%)	9.7%	−8.5–27.9%	0.299
Renal insufficiency ^, *n* (%)	62 (53.9%)	28 (46.7%)	34 (61.8%)	15.1%	−2.9–33.1%	0.103
eGFR (ml/min/1.73 m^2^), mean ± SD	60.7 ± 23.8	63.7 ± 20.7	57.4 ± 26.6	6.3	−2.5–15.1	0.159
Previous open-heart surgery						
Overall open-heart surgery, *n* (%)	41 (35.7%)	24 (40.0%)	17 (30.9%)	9.1%	−8.3–26.5%	0.309
CABG surgery, *n* (%)	13 (11.3%)	5 (8.3%)	8 (14.5%)	6.2%	−5.5–17.9%	0.293
Other surgery °, *n* (%)	8 (7.0%)	6 (10.0%)	2 (3.6%)	6.4%	−2.7–15.5%	0.180
VAD surgery, *n* (%)	22 (19.1%)	15 (25.0%)	7 (12.7%)	12.3%	−1.8–26.4%	0.095
Principal diagnosis for HTX						
Ischemic CMP, *n* (%)	32 (27.8%)	17 (28.3%)	15 (27.3%)	1.0%	−15.4–17.4%	0.899
Non-ischemic CMP, *n* (%)	63 (54.8%)	31 (51.7%)	32 (58.2%)	6.5%	−11.7–24.7%	0.483
Valvular heart disease, *n* (%)	3 (2.6%)	2 (3.3%)	1 (1.8%)	1.5%	−4.3–7.3%	0.611
Cardiac amyloidosis, *n* (%)	17 (14.8%)	10 (16.7%)	7 (12.7%)	4.0%	−8.9–16.9%	0.552
Donor data						
Age (years), mean ± SD	46.0 ± 11.8	46.4 ± 12.6	45.5 ± 11.0	0.9	−3.4– 5.2	0.663
Male sex, *n* (%)	44 (38.3%)	24 (40.0%)	20 (36.4%)	3.6%	−14.2–21.4%	0.689
BMI (kg/m^2^), mean ± SD	25.2 ± 4.3	25.6 ± 5.1	24.7 ± 3.1	0.9	−0.6–2.4	0.256
Transplant sex mismatch						
Mismatch, *n* (%)	47 (40.9%)	25 (41.7%)	22 (40.0%)	1.7%	−16.3–19.7%	0.856
Donor (m) to recipient (f), *n* (%)	3 (2.6%)	2 (3.3%)	1 (1.8%)	1.5%	−4.3–7.3%	0.611
Donor (f) to recipient (m), *n* (%)	44 (38.3%)	23 (38.3%)	21 (38.2%)	0.1%	−17.7–17.9%	0.987
Perioperative data						
Ischemic time (min), mean ± SD	253.9 ± 54.0	253.1 ± 57.5	254.9 ± 50.4	1.8	−17.9–21.5	0.858
Biatrial anastomosis, *n* (%)	1 (0.9%)	1 (1.7%)	0 (0.0%)	1.7%	−1.6–5.0%	0.336
Bicaval anastomosis, *n* (%)	114 (99.1%)	59 (98.3%)	55 (100.0%)	1.7%	−1.6–5.0%	0.336

BMI = body mass index; CABG = coronary artery bypass graft; CI = confidence interval; CMP = cardiomyopathy; COPD = chronic obstructive pulmonary disease; DOAC = direct oral anticoagulant; f = female; eGFR = estimated glomerular filtration rate; HTX = heart transplantation; m = male; *n* = number; OAC = oral anticoagulant; SD = standard deviation; VAD = ventricular assist device; VKA = vitamin K antagonist; ^ = eGFR < 60 mL/min/1.73 m^2^; ° = congenital, valvular, or ventricular surgery.

**Table 2:** Demographics—stratified by apixaban and rivaroxaban after HTX.

Parameter	Both DOACs after HTX (*n* = 55)	Apixaban after HTX (*n* = 27)	Rivaroxaban after HTX (*n* = 28)	Difference	95% CI	*p*-Value
Recipient data						
Age (years), mean ± SD	51.7 ± 11.1	52.6 ± 8.8	50.8 ± 13.1	1.8	−4.1–7.7	0.549
Male sex, *n* (%)	41 (74.5%)	20 (74.1%)	21 (75.0%)	0.9%	−22.1–23.9%	0.937
BMI (kg/m^2^), mean ± SD	25.2 ± 4.4	25.0 ± 4.3	25.4 ± 4.5	0.4	−1.9–2.7	0.736
Arterial hypertension, *n* (%)	27 (49.1%)	13 (48.1%)	14 (50.0%)	1.9%	−24.5–28.3%	0.891
Dyslipidemia, *n* (%)	30 (54.5%)	16 (59.3%)	14 (50.0%)	9.3%	−16.9–35.5%	0.491
Diabetes mellitus, *n* (%)	10 (18.2%)	6 (22.2%)	4 (14.3%)	7.9%	−12.4–28.2%	0.446
Peripheral artery disease, *n* (%)	2 (3.6%)	1 (3.7%)	1 (3.6%)	0.1%	−9.8–10.0%	0.979
COPD, *n* (%)	6 (10.9%)	3 (11.1%)	3 (10.7%)	0.4%	−16.1–16.9%	0.962
History of smoking, *n* (%)	29 (52.7%)	14 (51.9%)	15 (53.6%)	1.7%	−24.7–28.1%	0.898
Renal insufficiency ^, *n* (%)	26 (47.3%)	15 (55.6%)	11 (39.3%)	16.3%	−9.8–42.4%	0.227
eGFR (ml/min/1.73 m^2^), mean ± SD	63.8 ± 20.7	59.0 ± 21.3	68.3 ± 19.4	9.3	−1.5–20.1	0.097
Previous open-heart surgery						
Overall open-heart surgery, *n* (%)	21 (38.2%)	8 (29.6%)	13 (46.4%)	16.8%	−8.5–42.1%	0.200
CABG surgery, *n* (%)	5 (9.1%)	2 (7.4%)	3 (10.7%)	3.3%	−11.8–18.4%	0.670
Other surgery °, *n* (%)	5 (9.1%)	2 (7.4%)	3 (10.7%)	3.3%	−11.8–18.4%	0.670
VAD surgery, *n* (%)	13 (23.6%)	5 (18.5%)	8 (28.6%)	10.1%	−12.1–32.3%	0.380
Principal diagnosis for HTX						
Ischemic CMP, *n* (%)	17 (30.9%)	8 (29.6%)	9 (32.1%)	2.5%	−21.9–26.9%	0.840
Non-ischemic CMP, *n* (%)	28 (50.9%)	13 (48.1%)	15 (53.6%)	5.5%	−20.9–31.9%	0.688
Valvular heart disease, *n* (%)	1 (1.8%)	0 (0.0%)	1 (3.6%)	3.6%	−3.3–10.5%	0.322
Cardiac amyloidosis, *n* (%)	9 (16.4%)	6 (22.2%)	3 (10.7%)	11.5%	−7.9–30.9%	0.249
Donor data						
Age (years), mean ± SD	46.4 ± 12.0	47.6 ± 11.2	45.3 ± 12.8	2.3	−4.0–8.6	0.486
Male sex, *n* (%)	21 (38.2%)	8 (29.6%)	13 (46.4%)	16.8%	−8.5–42.1%	0.200
BMI (kg/m^2^), mean ± SD	25.7 ± 5.2	25.1 ± 4.6	26.3 ± 5.8	1.2	−1.5–3.9	0.384
Transplant sex mismatch						
Mismatch, *n* (%)	25 (45.5%)	12 (44.4%)	13 (46.4%)	2.0%	−24.3– 28.3%	0.883
Donor (m) to recipient (f), *n* (%)	2 (3.6%)	0 (0.0%)	2 (7.1%)	7.1%	−2.4–16.6%	0.157
Donor (f) to recipient (m), *n* (%)	23 (41.8%)	12 (44.4%)	11 (39.3%)	5.1%	−21.0–31.2%	0.698
Perioperative data						
Ischemic time (min), mean ± SD	251.4 ± 59.4	249.4 ± 53.2	253.3 ± 65.7	3.9	−27.7–35.5	0.812
Biatrial anastomosis, *n* (%)	1 (1.8%)	0 (0.0%)	1 (3.6%)	3.6%	−3.3–10.5%	0.322
Bicaval anastomosis, *n* (%)	54 (98.2%)	27 (100.0%)	27 (96.4%)	3.6%	−3.3–10.5%	0.322

BMI = body mass index; CABG = coronary artery bypass graft; CI = confidence interval; CMP = cardiomyopathy; COPD = chronic obstructive pulmonary disease; DOAC = direct oral anticoagulant; f = female; eGFR = estimated glomerular filtration rate; HTX = heart transplantation; m = male; *n* = number; SD = standard deviation; VAD = ventricular assist device; ^ = eGFR < 60 mL/min/1.73 m^2^; ° = congenital, valvular, or ventricular surgery.

**Table 3:** Medications—stratified by DOACs and VKAs after HTX.

Parameter	All OACs after HTX (*n* = 115)	DOACs after HTX (*n* = 60)	VKAs after HTX (*n* = 55)	Difference	95% CI	*p*-Value
Immunosuppressive drug therapy						
Cyclosporine A, *n* (%)	22 (19.1%)	11 (18.3%)	11 (20.0%)	1.7%	−12.7–16.1%	0.820
Tacrolimus, *n* (%)	73 (63.5%)	38 (63.3%)	35 (63.6%)	0.3%	−17.3–17.9%	0.973
Everolimus, *n* (%)	54 (47.0%)	28 (46.7%)	26 (47.3%)	0.6%	−17.7–18.9%	0.948
Azathioprine, *n* (%)	1 (0.9%)	0 (0.0%)	1 (1.8%)	1.8%	−1.7–5.3%	0.294
Mycophenolic acid, *n* (%)	80 (69.6%)	43 (71.7%)	37 (67.3%)	4.4%	−12.4–21.2%	0.609
Steroids, *n* (%)	55 (47.8%)	28 (46.7%)	27 (49.1%)	2.4%	−15.9–20.7%	0.795
Concomitant medications						
Oral antiplatelet drug, *n* (%)	18 (15.7%)	10 (16.7%)	8 (14.5%)	2.2%	−11.1–15.5%	0.754
Beta blocker, *n* (%)	76 (66.1%)	40 (66.7%)	36 (65.5%)	1.2%	−16.1–18.5%	0.891
Ivabradine, *n* (%)	30 (26.1%)	16 (26.7%)	14 (25.5%)	1.2%	−14.9–17.3%	0.882
Calcium channel blocker, *n* (%)	32 (27.8%)	17 (28.3%)	15 (27.3%)	1.0%	−15.4–17.4%	0.899
ACE inhibitor/ARB, *n* (%)	81 (70.4%)	43 (71.7%)	38 (69.1%)	2.6%	−14.1–19.3%	0.762
Diuretic, *n* (%)	82 (71.3%)	42 (70.0%)	40 (72.7%)	2.7%	−13.8–19.2%	0.747
Statin, *n* (%)	100 (87.0%)	53 (88.3%)	47 (85.5%)	2.8%	−9.6–15.2%	0.647
Gastric protection ^†^, *n* (%)	86 (74.8%)	44 (73.3%)	42 (76.4%)	3.1%	−12.8–19.0%	0.709

ACE inhibitor = angiotensin-converting-enzyme inhibitor; ARB = angiotensin II receptor blocker; CI = confidence interval; DOAC = direct oral anticoagulant; HTX = heart transplantation; *n* = number; OAC = oral anticoagulant; VKA = vitamin K antagonist; † = gastric protection drug defined as proton pump inhibitor (PPI) or histamine receptor (H2) blocker.

**Table 4:** Medications—stratified by apixaban and rivaroxaban after HTX.

Parameter	Both DOACs after HTX (*n* = 55)	Apixaban after HTX (*n* = 27)	Rivaroxaban after HTX (*n* = 28)	Difference	95% CI	*p*-Value
Immunosuppressive drug therapy						
Cyclosporine A, *n* (%)	8 (14.5%)	3 (11.1%)	5 (17.9%)	6.8%	−11.7–25.3%	0.478
Tacrolimus, *n* (%)	36 (65.5%)	19 (70.4%)	17 (60.7%)	9.7%	−15.3–34.7%	0.452
Everolimus, *n* (%)	28 (50.9%)	12 (44.4%)	16 (57.1%)	12.7%	−13.5–38.9%	0.346
Azathioprine, *n* (%)	0 (0.0%)	0 (0.0%)	0 (0.0%)	0.0%	n. a.	n. a.
Mycophenolic acid, *n* (%)	38 (69.1%)	20 (74.1%)	18 (64.3%)	9.8%	−14.5–34.1%	0.432
Steroids, *n* (%)	25 (45.5%)	11 (40.7%)	14 (50.0%)	9.3%	−16.9–35.5%	0.491
Concomitant medications						
Oral antiplatelet drug, *n* (%)	10 (18.2%)	5 (18.5%)	5 (17.9%)	0.6%	−19.8–21.0%	0.949
Beta blocker, *n* (%)	37 (67.3%)	16 (59.3%)	21 (75.0%)	15.7%	−8.8–40.2%	0.214
Ivabradine, *n* (%)	15 (27.3%)	6 (22.2%)	9 (32.1%)	9.9%	−13.4–33.2%	0.409
Calcium channel blocker, *n* (%)	15 (27.3%)	8 (29.6%)	7 (25.0%)	4.6%	−18.9–28.1%	0.700
ACE inhibitor/ARB, *n* (%)	39 (70.9%)	18 (66.7%)	21 (75.0%)	8.3%	−15.6–32.2%	0.496
Diuretic, *n* (%)	38 (69.1%)	17 (63.0%)	21 (75.0%)	12.0%	−12.3–36.3%	0.334
Statin, *n* (%)	49 (89.1%)	24 (88.9%)	25 (89.3%)	0.4%	−16.1–16.9%	0.962
Gastric protection ^†^, *n* (%)	39 (70.9%)	18 (66.7%)	21 (75.0%)	8.3%	−15.6–32.2%	0.496

ACE inhibitor = angiotensin-converting-enzyme inhibitor; ARB = angiotensin II receptor blocker; CI = confidence interval; DOAC = direct oral anticoagulant; HTX = heart transplantation; *n* = number; n. a. = not applicable; ^†^ = gastric protection drug defined as proton pump inhibitor (PPI) or histamine receptor (H2) blocker.

**Table 5:** Indications and complications—split by DOACs and VKAs after HTX.

Parameter	All OACs after HTX (*n* = 115)	DOACs after HTX (*n* = 60)	VKAs after HTX (*n* = 55)	Difference	95% CI	*p*-Value
Indications						
Atrial fibrillation, *n* (%)	33 (28.7%)	19 (31.7%)	14 (25.5%)	6.2%	−10.3–22.7%	0.462
Atrial flutter, *n* (%)	27 (23.5%)	16 (26.7%)	11 (20.0%)	6.7%	−8.7–22.1%	0.399
Pulmonary embolism, *n* (%)	8 (7.0%)	4 (6.7%)	4 (7.3%)	0.6%	−8.7–9.9%	0.898
Upper extremity DVT, *n* (%)	12 (10.4%)	6 (10.0%)	6 (10.9%)	0.9%	−10.3–12.1%	0.873
Lower extremity DVT, *n* (%)	28 (24.3%)	12 (20.0%)	16 (29.1%)	9.1%	−6.6–24.8%	0.257
Intracardiac thrombus, *n* (%)	7 (6.1%)	3 (5.0%)	4 (7.3%)	2.3%	−6.5–11.1%	0.611
OAC-related complications						
Overall bleedings, *n* (%)	24 (20.9%)	6 (10.0%)	18 (32.7%)	22.7%	8.2–37.2%	0.003 *
Ischemic stroke, *n* (%)	4 (3.5%)	2 (3.3%)	2 (3.6%)	0.3%	−6.4–7.0%	0.929
Thromboembolic event, *n* (%)	3 (2.6%)	2 (3.3%)	1 (1.8%)	1.5%	−4.2–7.2%	0.611
OAC-related death, *n* (%)	3 (2.6%)	1 (1.7%)	2 (3.6%)	1.9%	−4.0–7.8%	0.508
OAC-related bleedings						
Intracranial hemorrhage, *n* (%)	2 (1.7%)	0 (0.0%)	2 (3.6%)	3.6%	−1.3–8.5%	0.136
Severe epistaxis, *n* (%)	4 (3.5%)	1 (1.7%)	3 (5.5%)	3.8%	−3.1–10.7%	0.268
Gastrointestinal hemorrhage, *n* (%)	16 (13.9%)	4 (6.7%)	12 (21.8%)	15.1%	2.5–27.7%	0.019 *
Hemorrhagic shock, *n* (%)	2 (1.7%)	1 (1.7%)	1 (1.8%)	0.1%	−4.7–4.9%	0.950
Transfusion of FFP, *n* (%)	2 (1.7%)	1 (1.7%)	1 (1.8%)	0.1%	−4.7–4.9%	0.950
Transfusion of PRBCs, *n* (%)	22 (19.1%)	6 (10.0%)	16 (29.1%)	19.1%	4.9–33.3%	0.009 *

CI = confidence interval; DVT = deep vein thrombosis; DOAC = direct oral anticoagulant; FFP = fresh frozen plasma; HTX = heart transplantation; *n* = number; OAC = oral anticoagulant; PRBCs = packed red blood cells; VKA = vitamin K antagonist; * = statistically significant (*p* < 0.050).

**Table 6:** Indications and complications—split by apixaban and rivaroxaban after HTX.

Parameter	Both DOACs after HTX (*n* = 55)	Apixaban after HTX (*n* = 27)	Rivaroxaban after HTX (*n* = 28)	Difference	95% CI	*p*-Value
Indications						
Atrial fibrillation, *n* (%)	16 (29.1%)	10 (37.0%)	6 (21.4%)	15.6%	−8.1–39.3%	0.203
Atrial flutter, *n* (%)	16 (29.1%)	6 (22.2%)	10 (35.7%)	13.5%	−10.2–37.2%	0.271
Pulmonary embolism, *n* (%)	4 (7.3%)	3 (11.1%)	1 (3.6%)	7.5%	−6.2–21.2%	0.282
Upper extremity DVT, *n* (%)	5 (9.1%)	3 (11.1%)	2 (7.1%)	4.0%	−11.2–19.2%	0.609
Lower extremity DVT, *n* (%)	11 (20.0%)	4 (14.8%)	7 (25.0%)	10.2%	−10.7–31.1%	0.345
Intracardiac thrombus, *n* (%)	3 (5.5%)	1 (3.7%)	2 (7.1%)	3.4%	−8.5–15.3%	0.574
OAC-related complications						
Overall bleedings, *n* (%)	5 (9.1%)	3 (11.1%)	2 (7.1%)	4.0%	−11.2–19.2%	0.609
Ischemic stroke, *n* (%)	2 (3.6%)	1 (3.7%)	1 (3.6%)	0.1%	−9.8–10.0%	0.979
Thromboembolic event, *n* (%)	2 (3.6%)	1 (3.7%)	1 (3.6%)	0.1%	−9.8–10.0%	0.979
OAC-related death, *n* (%)	1 (1.8%)	1 (3.7%)	0 (0.0%)	3.7%	−3.4–10.8%	0.304
OAC-related bleedings						
Intracranial hemorrhage, *n* (%)	0 (0.0%)	0 (0.0%)	0 (0.0%)	0.0%	n.a.	n.a.
Severe epistaxis, *n* (%)	1 (1.8%)	1 (3.7%)	0 (0.0%)	3.7%	−3.4–10.8%	0.304
Gastrointestinal hemorrhage, *n* (%)	3 (5.5%)	1 (3.7%)	2 (7.1%)	3.4%	−8.5–15.3%	0.574
Hemorrhagic shock, *n* (%)	1 (1.8%)	1 (3.7%)	0 (0.0%)	3.7%	−3.4–10.8%	0.304
Transfusion of FFP, *n* (%)	1 (1.8%)	1 (3.7%)	0 (0.0%)	3.7%	−3.4–10.8%	0.304
Transfusion of PRBCs, *n* (%)	5 (9.1%)	3 (11.1%)	2 (7.1%)	4.0%	−11.2–19.2%	0.609

CI = confidence interval; DVT = deep vein thrombosis; DOAC = direct oral anticoagulant; FFP = fresh frozen plasma; HTX = heart transplantation; *n* = number; n.a. = not applicable; OAC = oral anticoagulant; PRBCs = packed red blood cells.

## Data Availability

The original contributions presented in this study are included in the article, further inquiries can be directed to the corresponding author.
